# On the Recombination Rate Estimation in the Presence of Population Substructure

**DOI:** 10.1371/journal.pone.0145152

**Published:** 2015-12-30

**Authors:** Julian Hecker, Dmitry Prokopenko, Christoph Lange, Heide Löhlein Fier

**Affiliations:** 1 Institute of Genomic Mathematics, University of Bonn, Bonn, Germany; 2 Department of Biostatistics, Harvard School of Public Health, Boston, United States of America; 3 Channing Laboratory, Brigham and Women’s Hospital, Boston, United States of America; 4 German Center for Neurodegenerative Diseases (DZNE), Bonn, Germany; Universitat Pompeu Fabra, SPAIN

## Abstract

As recombination events are not uniformly distributed along the human genome, the estimation of fine-scale recombination maps, e.g. HapMap Project, has been one of the major research endeavors over the last couple of years. For simulation studies, these estimates provide realistic reference scenarios to design future study and to develop novel methodology. To achieve a feasible framework for the estimation of such recombination maps, existing methodology uses sample probabilities for a two-locus model with recombination, with recent advances allowing for computationally fast implementations. In this work, we extend the existing theoretical framework for the recombination rate estimation to the presence of population substructure. We show under which assumptions the existing methodology can still be applied. We illustrate our extension of the methodology by an extensive simulation study.

## Introduction

The discovery that recombination events in human genome are not uniformly distributed, but concentrated in specific genomic regions, which are typically referred to as recombination hotspots [1], was one of the driving forces behind the HapMap Project [2]. The characterization and understanding of the local linkage disequilibrium structure between genetic variants is fundamental for the discovery of disease susceptibility loci (DSLs). The peak recombination rate within recombination hotspots can be multiple times higher than the recombination rate outside such genomic areas [3]. To create recombination hotspots in simulated data, commonly used software tools based on coalescent simulations, e.g. cosi, incorporate recombination rates that vary along the chromosome [4]. Such software tools, in order to generate realistic data, require an accurate fine-scale recombination rate map as input parameter. McVean et al. developed the LDhat software which estimates the recombination rate along the genome according to a assumed step-wise constant model [5]. The approach is computationally fast and can be applied at a genome-wide level. In 2007, Auton and McVean extended this approach and incorporated recombination hotspots into the assumed form of the recombination rate-model [6]. Both approaches are based on the so-called composite-likelihood approximation [7] that calculates the data likelihood as the product of pairwise two-locus probabilities. In order to obtain these sample probabilities, an Importance Sampling (IS) scheme by Fearnhead and Donnelly [8] was applied which requires exhaustive lookup tables of probabilities for a wide range of recombination rates. These methods were used to estimate the recombination rate for the HapMap samples CEU, YRI and ASN [2] (resp. later the 1000Genomes [9] samples), separately. As explained in [10], the overall genetic map of recombination rates was produced by comparison of the total map lengths to that estimated by the pedigree method in [11] and averaging these maps. In 2009, Jenkins and Song proposed approaches to calculate the sample probability for a given configuration with an analytic asymptotic formula of order two in the reciprocal recombination rate. Their approach was initially intended for an infinite-allele model [12] and later extended to a finite-allele model [13]. In particular, they showed how to transform the symmetric diallelic mutation model in Fearnhead and Donnelly [8] resp. LDhat to a so-called Parent-Independent-Mutation (PIM) model and were therefore able to present a different approach to calculate the required two-locus probabilities for LDhat. The key aspect they showed was that the calculations were independent from the specific value of the recombination rate. Thus, they were able to evaluate the sample probability instantly for every recombination rate. This substantially reduces the computational burden. In addition, for large recombination rate values, Monte Carlo based methods as the IS scheme by Fearnhead and Donnelly are less efficient, since the number of recombination events increases and the sampled genealogies become very complicated, whereas the asymptotic sampling formula by Jenkins and Song becomes very precise for large values. In 2012, Jenkins and Song derived an arbitrary order asymptotic expansion for the finite-allele model, using the generator of the corresponding Wright-Fisher diffusion [14]. Together with the application of Pade approximations, this provided a way to evaluate two-locus probabilities efficiently for a wide range of recombination rates. The mentioned diffusion generator used in [14] describes the diffusion limit of the Markov Chain explained in [15]. In this communication, we extend this underlying Markov Chain model to the presence of population substructure with a finite number of subpopulations. Under the assumption of strong migration between the subpopulations, we derive the corresponding diffusion limit of a specific weighted mean of subpopulation frequencies. The key result of our work is, that differences between subpopulation frequencies disappear and the diffusion limit has the form as in the panmictic case with rescaled effective population size. This implies the possibility to combine subpopulation samples and evaluate the corresponding sample probability with the existing methodology without any additional computation effort. The advantage for the estimation of recombination rates lies in the increased sample size and the more realistic underlying model.

## Methods

We start with a summary of the results in [13]. We denote the sample, i.e. the genetic data at locus A and B for *n* study subjects, by **s**. The parameter *ρ* = 4*N*_*e*_ *r* is the population-scaled recombination rate. The parameters *θ*_*A*_ and *θ*_*B*_ refer to the mutation rates at locus A and B. Let *r*_*A*_ be the number of possible alleles at the first locus *A* and *r*_*B*_ be the number of possible alleles at the other locus *B*. Denote the different alleles by *A*_1_, …, *A*_*r*_*A*__ and *B*_1_, …, *B*_*r*_*B*__. For notational convenience, we introduce [*k*]: = {1, …, *k*}. First, we summarize the work in [14] to emphasize the differences to our model later. Write the sample configuration **s** for a single population by

$$\begin{array}{c} s=(a,b,c) \end{array}$$

with

$$\begin{array}{c} c={\left(c_{ij}\right)}_{i\in \left[r_{A}\right],j\in \left[r_{B}\right]}, \end{array}$$

where *c*_*ij*_ gives the number of gametes with allele *A*_*i*_ at locus *A* and *B*_*j*_ at locus *B*. Similar,

$$\begin{array}{c} a=(a_{1},\cdots ,a_{r_{A}}), \end{array}$$

where *a*_*i*_ gives the number of gametes with allele *A*_*i*_ at locus *A* and unspecified allele at locus *B*. **b** is defined analogously. Further, write $a=\sum_{i=1}^{r_{A}}a_{i}$, $b=\sum_{j=1}^{r_{B}}b_{j}$, $c=\sum_{i=1}^{r_{A}}\sum_{j=1}^{r_{B}}c_{ij}$ and *n* = *a* + *b* + *c*. This notation is also used in [13]. Jenkins and Song utilize the fact that, under a stationary distribution, the expectation of a suitable function, which is applied to the generator (described in [14]), is equal to zero. If *q*(**s**; *ρ*) describes the sample probability with reference to *ρ*, they derived that the sample probabilities can be calculated from the following linear system

$$\begin{array}{c} \left[n\left(n-1\right)+\theta_{A}\left(a+c\right)+\theta_{B}\left(b+c\right)+\rho c\right]q\left(\left(a,b,c\right);\rho \right)=\\ \sum_{i=1}^{r_{A}}a_{i}\left(a_{i}-1+2c_{i•}\right)q\left(\left(a-e_{i},b,c\right);\rho \right)+\sum_{j=1}^{r_{B}}b_{j}\left(b_{j}-1+2c_{•j}\right)q\left(\left(a,b-e_{j},c\right);\rho \right)\\ +\sum_{i=1}^{r_{A}}\sum_{j=1}^{r_{B}}\left[c_{ij}\left(c_{ij}-1\right)q\left(\left(a,b,c-e_{ij}\right);\rho \right)+2a_{i}b_{j}q\left(\left(a-e_{i},b-e_{j},c+e_{ij}\right);\rho \right)\right]\\ +\theta_{A}\sum_{i=1}^{r_{A}}\left[\sum_{j=1}^{r_{B}}c_{ij}\sum_{k=1}^{r_{A}}P_{ki}^{A}q\left(\left(a,b,c-e_{ij}+e_{kj}\right);\rho \right)+a_{i}\sum_{k=1}^{r_{A}}P_{ki}^{A}q\left(\left(a-e_{i}+e_{k},b,c\right);\rho \right)\right]\\ +\theta_{B}\sum_{j=1}^{r_{B}}\left[\sum_{i=1}^{r_{A}}c_{ij}\sum_{l=1}^{r_{B}}P_{lj}^{B}q\left(\left(a,b,c-e_{ij}+e_{il}\right);\rho \right)+b_{j}\sum_{l=1}^{r_{B}}P_{lj}^{B}q\left(\left(a,b-e_{j}+e_{l},c\right);\rho \right)\right]\\ +\rho \sum_{i=1}^{r_{A}}\sum_{j=1}^{r_{B}}c_{ij}q\left(\left(a+e_{i},b+e_{j},c-e_{ij}\right);\rho \right), \end{array}$$ (1)

with boundary conditions

$$\begin{array}{c} q\left(\left(e_{i},0,0\right);\rho \right)=\pi_{i}^{A}\;\text{and}\;q\left(\left(0,e_{j},0\right);\rho \right)=\pi_{j}^{B}\;\text{for}\;\text{all}\;i\in \left[r_{A}\right],j\in \left[r_{B}\right], \end{array}$$

where *π* ^*A*^ and *π* ^*B*^ denote the stationary distributions of the mutation models for locus *A* and *B*. Since this linear system has to be solved for every recombination rate *ρ* separately and grows rapidly in *n*, they propose an asymptotic expansion in $\frac{1}{\rho }$

$$\begin{array}{c} q\left(s;\rho \right)=q_{0}\left(s\right)+\frac{q_{1}\left(s\right)}{\rho }+\frac{q_{2}\left(s\right)}{\rho^{2}}+\cdots . \end{array}$$

Now, the corresponding recursions are solved only once for a fixed mutation model and the sample probabilities, with reference to several recombination rates, can be evaluated by plugging in the recombination parameter *ρ*. The estimation procedures in [5] and [6] are based on two-locus probabilities from the model in [8] with respect to some mutation parameter *θ*_*FD*_, recombination rate *ρ* and a symmetric diallelic mutation model

$$\begin{array}{c} P_{FD}=\left(\begin{array}{cc} 0 & 1\\ 1 & 0 \end{array}\right). \end{array}$$

As shown in [13], both models are in line if we set *r*_*A*_ = *r*_*B*_ = 2, *θ* = 2*θ*_*FD*_ and

$$\begin{array}{c} P=\left(\begin{array}{cc} \frac{1}{2} & \frac{1}{2}\\ \frac{1}{2} & \frac{1}{2} \end{array}\right). \end{array}$$

As explained in the introduction, the generator in [14] corresponds to the diffusion limit of the discrete Markov Chain in [15]. In the next section, we introduce the population substructured extension of this discrete Markov Chain, derive the diffusion limit of weighted mean frequencies and show that we can utilize the same idea as in [14] to improve the recombination rate estimation framework.

### Model setting

As explained, we consider a two-locus model. As in [14], let *r*_*A*_ be the number of possible alleles at the first locus *A* and *r*_*B*_ be the number of possible alleles at the other locus *B*. The generations are supposed to be non-overlapping and the population is monoecious. We suppose the underlying model for the discrete Wright-Fisher model as it is described in [15], which leads to a diffusion approximation, corresponding to the generator in [14]. But in addition, we assume that the population is subdivided into Γ < ∞ subpopulations. Denote by $q_{\alpha }^{{}^{\prime }}$, *α* = 1, …, Γ, the fractions of the corresponding subpopulation sizes and let *N* be the overall population size. Thus, $0<q_{\alpha }^{{}^{\prime }}<1$ and $\sum_{\alpha =1}^{\Gamma }q_{\alpha }^{{}^{\prime }}=1$. Set $N_{\alpha }=q_{\alpha }^{{}^{\prime }}N$ for *α* = 1, …, Γ. We model the migration procedure as in the discrete model in [16]. In this context, let *m*_*αβ*_ denote the probability that an individual in subpopulation *α* was in subpopulation *β* one generation before. These probabilities are given by the backward migration matrix *M*. The following diagram summarizes the lifecycle of the model (compare with [16]).

$$\begin{array}{c} \begin{array}{ccccccc} \text{Adult} & \overset{\text{reproduction}}{\to } & \text{Zygote} & \overset{\text{recombination}}{\to } & \text{Adult} & \overset{\text{migration}}{\to } & \text{Adult}\\ {\left(N_{\alpha },{\left(P_{\alpha ij}\right)}_{ij}\right)}_{\alpha } & & {\left(\infty ,{\left(P_{\alpha ij}\right)}_{ij}\right)}_{\alpha } & & {\left(\infty ,{\left(P_{\alpha ij}^{*}\right)}_{ij}\right)}_{\alpha } & & {\left(\infty ,{\left(P_{\alpha ij}^{**}\right)}_{ij}\right)}_{\alpha } \end{array}\\ \begin{array}{cccc} \overset{\text{mutation}}{\to } & \text{Adult} & \overset{\text{regulation}}{\to } & \text{Adult}\\ & {\left(\infty ,{\left(P_{\alpha ij}^{***}\right)}_{ij}\right)}_{\alpha } & & {\left(\infty ,{\left(P_{\alpha ij}^{\prime }\right)}_{ij}\right)}_{\alpha } \end{array} \end{array}$$

The Markov Chain *Z* ^*N*^, which describes the relative frequencies (*P*_*αij*_)_*α*, *i*, *j*_, is completely defined in S1 Appendix. *P*_*αij*_ denotes the relative frequency of type *A*_*i*_ *B*_*j*_ in subpopulation *α* in the model *Z* ^*N*^, we suppress the dependency on *N* for convenience. Note that the subpopulation sizes are assumed to be effectively infinite except shorlty before and after population regulation [16].

### Parameter assumptions

In order to obtain a diffusion limit, we make the following assumptions on the model parameters.

#### Biological parameters

We use the well established assumptions on the parameter scaling for the biological mechanisms. These conditions depend on the effective population size (section 3.7 in [17]). The effective population size for our model is derived below. We write

$$\begin{array}{c} u_{ki}^{N}=u_{A}^{N}P_{ki}^{A}>0,\;\text{for}\;\text{all}\;i,k\in \left[r_{A}\right],i\neq k, \end{array}$$ (2)

and

$$\begin{array}{c} v_{jl}^{N}=u_{B}^{N}P_{jl}^{B}>0,\;\text{for}\;\text{all}\;j,l\in \left[r_{B}\right],j\neq l, \end{array}$$ (3)

where *u*_*A*_ and *u*_*B*_ are the mutation rates per individual per generation and *P* ^*A*^ as well as *P* ^*B*^ are the transition matrices for mutation for location *A* resp. *B* with $P_{ii}^{A}>0$ and $P_{jj}^{B}>0$ as in [14]. See Eq (A.1) and (A.2) in S1 Appendix for the definition of the mutation model. For convenience, we omit technical conditions of the truncation *N*, since we are only interested in the diffusion limit resp. large *N*. We assume

$$\begin{array}{c} \lim_{N\to \infty }4N_{e}u_{A}^{N}=\theta_{A} \end{array}$$

resp.

$$\begin{array}{c} \lim_{N\to \infty }4N_{e}u_{B}^{N}=\theta_{B}. \end{array}$$

For the recombination fractions in Eq (A.3) resp. (A.4) in S1 Appendix, we suppose

$$\begin{array}{c} \lim_{N\to \infty }4N_{e}r_{N}=\rho <\infty . \end{array}$$ (4)

#### Migration

The backward migration matrix *M* is assumed to be a stochastic matrix, which is irreducible and aperiodic. Migration is independent of time and *N*_*e*_ (see below) resp. *N*. This is the strong migration assumption. Denote the stationary distribution of *M* by *ξ*. This means that migration dominates all other evolutionary forces. This assumption was used in several papers. We adopted it from [16]. Additionally, this suggestion was analyzed in [18], in other papers of [19–21] and [22–24].

### Diffusion approximation

The next step is to analyze the diffusion limit of the Markov Chain *Z* ^*N*^. As in [15] and [16], this is essentially an application of the diffusion approximation Theorem in [25] in S1 Appendix, which is also stated as Theorem (A.3). We describe by simple calculation that the Theorem is still applicable for our extended Markov Chain S1 Appendix. Important for the derivation of the diffusion limit is the connection between *N* and *N*_*e*_, the effective population size. Nagylaki observed and described this connection in the setting of a subdivided population with strong migration in a one-locus model in [16]. He stated

$$\begin{array}{c} N_{e}=\delta N, \end{array}$$

where

$$\begin{array}{c} \delta ={\left[\sum_{\alpha }\xi_{\alpha }^{2}/q_{\alpha }^{{}^{\prime }}\right]}^{-1} \end{array}$$ (5)

Therefore, *N*_*e*_ ≤ *N* and in particular *N*_*e*_ is equal to *N* if and only if $\sum_{\alpha }q_{\alpha }^{{}^{\prime }}m_{\alpha \beta }=q_{\beta }$. This scenario is called conservative migration. These results are still valid in our two-locus scenario. Analogous to [16], we define

$$\begin{array}{c} X^{N}:=\Phi_{N}\left({\left(P_{\alpha ij}\right)}_{\alpha ij}\right)={\left(P_{ij}\right)}_{(i,j)\in J}=:P, \end{array}$$ (6)

where $P_{ij}=\sum_{\alpha }\xi_{\alpha }P_{\alpha ij}$ and

$$\begin{array}{c} Y^{N}:=\Psi_{N}\left({\left(P_{\alpha ij}\right)}_{\alpha ij}\right)={\left(d_{\alpha ij}\right)}_{\alpha ,(i,j)\in J}=:d, \end{array}$$ (7)

where *d*_*αij*_ = *P*_*αij*_ − *P*_*ij*_. In addition,

$$\begin{array}{c} J:=\{\left(i,j\right):i\in \left\{1,\cdots ,r_{A}\right\},j\in \left\{1,\cdots ,r_{B}\right\},\left(i,j\right)\neq \left(r_{A},r_{B}\right)\}. \end{array}$$ (8)

The idea is to set the two timescales in Theorem (A.3) to *ε*_*N*_ = 2*N*_*e*_ and *δ*_*N*_ = 1. Recombination and mutation work on the slow timescale *ε*_*N*_, but the strong migration assumption implies that migration works on the fast timescale *δ*_*N*_. Then, it follows that the scaled *X* ^*N*^([2*N*_*e*_•]) converges to a diffusion process *X* with explicitly known generator (A.17). The process *Y* ^*N*^ describes the derivation of the frequencies within the subpopulations from the weighted mean frequencies. The result is, that *Y* ^*N*^([2*N*_*e*_ *t*]]) converges to 0, for every *t* > 0. The interpretation is that, since migration works faster than the biological mechanisms, the differences in relative frequencies within the subpopulations disappear and approach the mean frequencies. In S1 Appendix, it is shown that the process *X* ^*N*^[2*N*_*e*_•] converges weakly to a process *X* with state space (A.16), which is associated to the generator (A.17) resp. (A.23) with state space (A.22). This generator has the same form as the generator which was used in [14]. The difference lies in the scaled effective population size. This observation is in agreement with the one-locus results in [16]. The assumptions about the mutation model (Eqs (2) and (3)) imply an important fact about the discrete Markov Chains.

**Lemma 1**: The Markov chain *Z* ^*N*^ has a unique stationary distribution *μ*_*N*_.

*Proof* See S1D Appendix.

Another important observation is that there exists a unique stationary distribution *μ* for the diffusion process *X*.

**Lemma 2**: The diffusion process *X* has a unique stationary distribution *μ*.

*Proof* This is a consequence of the restriction of the results in [26] to a finite number of possible alleles.

### Sample configurations

As described in the introduction, the main interest lies in the efficient evaluation of approximate sample probabilities. Therefore, we need to extend the definition of the latter object to incorporate our scenario. A sample configuration for a subdivided population is defined by

$$\begin{array}{c} s=(s_{1},\cdots ,s_{\Gamma }), \end{array}$$

where

$$\begin{array}{c} s_{\alpha }=\left(a_{\alpha },b_{\alpha },c_{\alpha }\right)\;\text{for}\;\alpha \in \left[\Gamma \right]. \end{array}$$

Here, we have

$$\begin{array}{c} c_{\alpha }={\left(c_{\alpha ij}\right)}_{i\in \left[r_{A}\right],j\in \left[r_{B}\right]}, \end{array}$$

where *c*_*αij*_ gives the number of gametes in subpopulation *α* with allele *A*_*i*_ at locus *A* and *B*_*j*_ at locus *B*. Similar,

$$\begin{array}{c} a_{\alpha }=\left(a_{\alpha 1},\cdots ,a_{\alpha r_{A}}\right), \end{array}$$

where *a*_*αi*_ gives the number of gametes in subpopulation *α* with allele *A*_*i*_ at locus *A* and unspecified allele at locus *B*. **b**_*α*_ is defined analogously. This is the straightforward extension of the definition in [14].

### Sample probabilities

Recall the stationary distributions *μ*_*N*_ of the discrete model and define

$$\begin{array}{c} P_{\Gamma }\left({\left(P_{\alpha ij}\right)}_{\alpha ij};s\right)=\prod_{\alpha =1}^{\Gamma }P\left({\left(P_{\alpha ij}\right)}_{ij};s_{\alpha }\right) \end{array}$$

with

$$\begin{array}{c} P\left({\left(P_{\alpha ij}\right)}_{ij};s_{\alpha }\right)=\left(\prod_{i}P_{\alpha i•}^{a_{\alpha i}}\right)\left(\prod_{j}P_{\alpha •j}^{b_{\alpha j}}\right)\left(\prod_{i,j}P_{\alpha ij}^{c_{\alpha ij}}\right),\;\alpha \in \left[\Gamma \right], \end{array}$$

where $P_{\alpha i•}=\sum_{j=1}^{r_{B}}P_{\alpha ij}$ and *P*_*α*•*j*_ analogously. The probability of a sample configuration for the model can be expressed as

$$\begin{array}{c} \mu_{N}\left(P_{\Gamma }\left({\left(P_{\alpha ij}\right)}_{\alpha ij};s\right)\right). \end{array}$$

If we have Γ = 1, this is in line with the approach in [14]. Based on the observations above, we can derive that we can approximate these sample probabilities by combined samples for the diffusion limit. More precise:

**Lemma 3**:

$$\begin{array}{c} \lim_{N\to \infty }\mu_{N}\left(P_{\Gamma }\left({\left(P_{\alpha ij}\right)}_{\alpha ij};s\right)\right)=\mu \left(P({\left(x_{ij}\right)}_{ij};\cup_{\alpha }s_{\alpha })\right), \end{array}$$

where ∪_*α*_ **s**_*α*_ is the combined sample for one population with $a_{i}=\sum_{\alpha =1}^{\Gamma }a_{\alpha i}$, $b_{j}=\sum_{\alpha =1}^{\Gamma }b_{\alpha j}$ and $c_{ij}=\sum_{\alpha =1}^{\Gamma }c_{\alpha ij}$ for all *i*, *j*.

*Proof*. See S1E Appendix.

*μ* is the stationary distribution of the diffusion process *X* and (*x*_*ij*_)_*ij*_ in the state space *K*_*c*_ (A.22). We do not distinguish in the notation between these objects in different characterizations, since they can be identified with each other.

#### Main result

Thus, our main result is that we can combine the samples over all subpopulations and evaluate the corresponding sample probability for the diffusion process, which is stated above. The advantage is that the computational effort is still the same as in the panmictic case. The only adjustment is done by the rescaling of the effective population size. To clarify this connection, note that the generator for a single population in [14], has the same form and depends on the recombination rate *ρ* and mutation rates *θ*_*A*_ resp. *θ*_*B*_. As mentioned above, Jenkins and Song evaluated the expression for the sample probability, given biological parameters, with a sophisticated recursion technique, which was derived by the fact that

$$\begin{array}{c} \mu (LP)=0. \end{array}$$

Here denotes L the associated generator of the diffusion process. For our scenario, we are dealing with a rescaled effective population size, conditioned on the migration model, by the factor *δ* in Eq (5) and can apply the same technique.

## Results/Simulation study

In order to give a better impression about the results, we present an empirical example for the theory. We consider a model with two subpopulations. A realistic choice for *N* is about *N* = 10,000, according to [4]. The fractions of subpopulation sizes are denoted by $q_{1}^{{}^{\prime }}$ and $q_{2}^{{}^{\prime }}$. We choose the backward migration matrix to be equal to

$$\begin{array}{c} M=\left(\begin{array}{cc} 0.9 & 0.1\\ 0.2 & 0.8 \end{array}\right). \end{array}$$

Note that this matrix satisfies the migration assumptions and implies $\xi =(\frac{2}{3},\frac{1}{3})$. We consider a diallelic model for both loci, i.e.

$$\begin{array}{c} r_{A}=2,\;r_{B}=2, \end{array}$$

and assume that the mutation rates are the same for loci *A* and loci *B*. Furthermore, we choose

$$\begin{array}{c} P^{A}=P^{B}=\left(\begin{array}{cc} 0.5 & 0.5\\ 0.5 & 0.5 \end{array}\right), \end{array}$$

as in [14]. As it is used in [14] (as explained in the Methods section, this corresponds to the mutation model used in LDhat), reasonable parameter values for *θ* are *θ*_*A*_ = *θ*_*B*_ = *θ* ∈ [0.001, 0.01] and *ρ* = 50. To test our results, we simulate the discrete model *Z* ^*N*^ of the relative frequencies in both subpopulations with arbitrary initial distributions according to Eqs (A.4), (A.5), (A.6) and (A.7) in S1 Appendix. For this, we compute the corresponding mutation and recombination rates *u*_0_ and *r*_0_ satisfying

$$\begin{array}{c} \theta =4N_{e}u_{0} \end{array}$$

and

$$\begin{array}{c} \rho =4N_{e}r_{0}. \end{array}$$

for *θ* = 0.01, *ρ* = 50 and *N*_*e*_ = 10,000, to get realistic parameter values for the Markov Chain. To obtain an estimate of the sample probability under the stationary distribution, we run the model with respect to *u*_0_ and *r*_0_ over 10^12^ generations and estimate the corresponding quantity for some example configurations every 10^5^ generations. We do this for two different scenarios of subpopulation sizes. The first choice is (*q*_1_, *q*_2_) = *ξ* and the second $\left(q_{1},q_{2}\right)=\left(\frac{1}{5},\frac{4}{5}\right)$. In the first case, the condition for conservative migration is satisfied, *δ*_1_ = 1. Therefore, the theory predicts that the effects of the subdivision disappear. In the second scenario, we compute

$$\begin{array}{c} \delta_{2}={\left(\frac{{\left(\frac{2}{3}\right)}^{2}}{\frac{1}{5}}+\frac{{\left(\frac{1}{3}\right)}^{2}}{\frac{4}{5}}\right)}^{-1}\approx \frac{1}{2.3611}. \end{array}$$

Based on these observations, we compare the empirical results for the discrete model with the results from the diffusion approximation. As explained, we have to take care about the corresponding scaled mutation and recombination rates. In the first case, this implies *θ* = 0.01 and *ρ* = 50, since *δ*_1_ = 1, and in the second case *θ* ≈ 0.004235 and *ρ* ≈ 21.17. The sample probabilities for the diffusion approximation can either be obtained by the software package asf [13] or can be calculated by solving the linear system Eq (1). Solving the linear system gives us the exact value for the sample probabilities, the software asf only calculates a second order approximation. We implement a solver for the linear system, similar to the goldings recursion program from Richard R. Hudson [7], and use these results (the results are consistent with the the sample probabilities from asf). The comparison of the values is given in the Tables 1 and 2. We only consider sample configurations with full gamete information, e.g. *a*_1_ = *a*_2_ = *b*_1_ = *b*_2_ = 0. As we can see in the Tables 1 and 2, the observations are in line with the theoretical predictions.

**Table 1:** Comparison of estimated and expected probabilities for the scenario (*q*_1_, *q*_2_) = (*ξ*_1_, *ξ*_2_).

sample configuration	discrete	combined sample	diffusion
((0, 0, 1, 0), (0, 0, 0, 1))	0.00123218	(0, 0, 1, 1)	0.00123137
((0, 0, 1, 1), (0, 0, 1, 0))	0.000615191	(0, 0, 2, 1)	0.00061418
((1, 0, 2, 0), (1, 0, 2, 0))	6.1255e-05	(2, 0, 4, 0)	6.1262e-05
((4, 0, 0, 0), (2, 0, 0, 0))	0.245318	(6, 0, 0, 0)	0.244383
((3, 0, 0, 0), (2, 1, 0, 0))	0.000243998	(5, 1, 0, 0)	0.000244123
((1, 1, 0, 0), (0, 0, 0, 1))	1.46358e-06	(1, 1, 0, 1)	1.50463e-06

6 different example sample configurations. The discrete value corresponds to the estimated probability from the discrete model, the diffusion value to exact solution of the linear system.

**Table 2:** Comparison of estimated and expected probabilities for the scenario $\left(q_{1},q_{2}\right)=\left(\frac{1}{5},\frac{4}{5}\right)$.

sample configuration	discrete	combined sample	diffusion
((0, 0, 1, 0), (0, 0, 0, 1))	0.000522292	(0, 0, 1, 1)	0.000525972
((0, 0, 1, 1), (0, 0, 1, 0))	0.000260697	(0, 0, 2, 1)	0.00026271
((1, 0, 2, 0), (1, 0, 2, 0))	2.62979e-05	(2, 0, 4, 0)	2.62440e-05
((4, 0, 0, 0), (2, 0, 0, 0))	0.248091	(6, 0, 0, 0)	0.247599
((3, 0, 0, 0), (2, 1, 0, 0))	0.000104652	(5, 1, 0, 0)	0.000104806
((1, 1, 0, 0), (0, 0, 0, 1))	2.60423e-07	(1, 1, 0, 1)	2.67692e-07

Description: See Table 1.

## Discussion

In their work, Jenkins and Song derived an approximate sampling formula of arbitrary order from the generator of the Wright-Fisher diffusion [14]. Relying on first results in [13] and the detailed study of accuracy in [14], we restate that the application of the approximate sampling formula has huge advantages in relation to the computational demanding Monte Carlo based methods, especially for large values of *ρ*. These theoretical results can be applied to make the existing recombination rate estimation procedures as implemented in LDhat and rhomap more efficient. In this communication, we go one step further and extend the underlying discrete Wright-Fisher model to the scenario in which the sample population consists of distinct subpopulations. We describe the required assumptions for the biological parameters and the migration model, in order to derive the corresponding diffusion limit of the weighted mean frequencies. The strong migration assumption implies that the form of the generator for the diffusion limit is the same as in the panmictic case. Under our mutation and recombination model, we can show the existence and uniqueness of stationary distributions for the discrete Markov Chains and the diffusion process. We describe the connection between these stationary distributions, which is essentially the motivation for the approach to compute approximate sample probabilities. An empirical example shows that our theoretical derivations are valid. The conclusion is that the probability of a sample containing subsamples from each subpopulation can be calculated as the probability of the combined sample with the original approach of [14]. The difference lies in the rescaled effective population size. The advantage is that one can handle 2-loci data for multiple subpopulations without any additional computational burden.

We believe that the suggested extension can help to achieve a more realistic setting for the recombination rate estimation and increased efficiency due to larger sample sizes.

## Supporting Information

S1 Appendix Theoretical derivations and description. In this file, we describe the underlying Markov Chain, the theoretical tools behind the diffusion limit and the proofs of Lemma 1 and 3.
(PDF)
