# Supplementary material for: On the Recombination Rate Estimation in the Presence of Population Substructure
Source: PLoS One. 2015 Dec 30;10(12):e0145152. doi: 10.1371/journal.pone.0145152 (PMC4696844; doi:10.1371/journal.pone.0145152)
Supplement: S1 Appendix — In this file, we describe the underlying Markov Chain, the theoretical tools behind the diffusion limit and the proofs of Lemma 1 and 3. (PDF) [file pone.0145152.s001.pdf]

A S1 Appendix

A Description of the discrete Markov Chain

We describe a single transition of the Markov Chain. For convenience, we drop the dependency on the parameter N for the relative frequencies $P_{\alpha ij}$. Denote the mutation probability for a mutation at locus A from allele A_k to A_i by u_{ik}. Analogously, v_{jl} denotes the mutation probability for locus B. For technical reasons, we set $u_{ii} = 0$ and $v_{jj} = 0$ and write

$$u_{ki}^{N*} = \left(1 - \sum_{m}^{r_A} u_{im}^{N}\right) \delta_{ik} + u_{ik}, \tag{A.1}$$

as well as

$$v_{jl}^{N*} = \left(1 - \sum_{n}^{r_B} v_{jn}^{N}\right) \delta_{jl} + v_{jl}. \tag{A.2}$$

for fixed N. Let $0 \leq r_N \leq \frac{1}{2}$ denote the fraction of recombination. Therefore, we get the term

$$\theta_{ij;kl,mn}^{N} = \frac{1}{2}\delta_{ik}[(1 - r_N)\delta_{jl} + r_N\delta_{jn}] + \frac{1}{2}\delta_{im}[(1 - r_N)\delta_{jn} + r_N\delta_{jl}] \tag{A.3}$$

as the probability to draw a A_iB_j gamete from an A_kB_l/A_mB_n gamete. The Markov chain Z^N in

$$E_N := K_{N_1} \times \cdots \times K_{N_\Gamma}$$

is defined as follows. Here we have

$$K_{N_\alpha} = \{N_\alpha^{-1}\beta : \beta \in (\mathbb{Z}_+)^{r_A r_B}, \sum_{i=1}^{r_A}\sum_{j=1}^{r_B} \beta_{ij} = N_\alpha\}.$$

The equations for the corresponding steps in the life cycle can be written as

$$P_{\alpha ij}^{*} = \sum_{k,l,m,n} \theta_{ij;kl,mn}^{N} P_{\alpha kl} P_{\alpha mn}, \tag{A.4}$$

$$P_{\alpha ij}^{**} = \sum_{\beta} m_{\alpha\beta} P_{\beta ij}^{*}, \tag{A.5}$$

and

$$P_{\alpha ij}^{***} = \sum_{k,l} u_{ki}^{*N} v_{lj}^{*N} P_{\alpha kl}^{**}. \tag{A.6}$$

The last step is formulated as

$$P_{\alpha ij}^{'} \sim (2N_\alpha)^{-1}\text{multinomial}(2N_\alpha, (P_{\alpha ij}^{***})_{ij}). \tag{A.7}$$

As mentioned, this Markov Chain is a combination of the two-locus Markov Chain in [1] and the one-locus migration model in [2].

B Diffusion approximation

The following statements are pure restatements of the results in [3], chapter 10.

Introduce two compact, convex subsets K and H of $\mathbb{R}^m$ and $\mathbb{R}^n$, respectively, with nonempty interior. Assume that $0 \in H$. Furthermore, we call a Strong Continuous Contraction Semigroup in the following SCCSG.

Lemma A.1. *Let $c : K \times \mathbb{R}^n \to \mathbb{R}^n$ be of class C^2 and such that the solution $Y(t, x, y)$ of the differential equation*

$$\frac{d}{dt} Y(t, x, y) = c(x, Y(t, x, y)), \quad Y(0, x, y) = y,$$

exists for all $(t, x, y) \in [0, \infty) \times K \times H$ and satisfies

$$\lim_{t \to \infty} \sup_{(x,y) \in K \times H} |Y(t, x, y)| = 0.$$

Then there exists a compact set E, with $K \times H \subset E \subset K \times \mathbb{R}^n$, such that $(x, y) \in E$ implies $(x, Y(t, x, y)) \in E$ for all $t \geq 0$, and the formula

$$S(t)h(x, y) = h(x, Y(t, x, y))$$

defines a SCCSG on $C(E)$(with sup norm). The generator B of $S(t)$ has $C^2(E) = \{f|_E : f \in C^2(\mathbb{R}^m \times \mathbb{R}^n)\}$ as a core and the form

$$Bh(x, y) = \sum_{l=1}^{n} c_l(x, y) \frac{\partial}{\partial y_l} h(x, y) \quad \text{on } K \times H, \quad h \in C^2(E).$$

Finally,

$$\lim_{t \to \infty} \sup_{(x,y) \in E} |S(t)h(x, y) - h(x, 0)| = 0, \quad h \in C(E).$$

Lemma A.2. *Given $\delta_\infty > 0$, let $c : K \times \mathbb{R}^n \to \mathbb{R}^n$ be continuous, such that the solution $Y(k, x, y)$ of the difference equation*

$$\delta_\infty^{-1}(Y(k + 1, x, y) - Y(k, x, y)) = c(x, Y(k, x, y)), \quad Y(0, x, y) = y,$$

which exits for all $(k, x, y) \in \mathbb{Z}_+ \times K \times H$, satisfies

$$\lim_{k \to \infty} \sup_{(x,y) \in K \times H} |Y(k, x, y)| = 0.$$

Then there exists a compact set E, with $K \times H \subset E \subset K \times \mathbb{R}^n$, such that $(x, y) \in E$ implies $(x, Y(k, x, y)) \in E$ for $k = 0, 1, \ldots,$ and the formula

$$S(t)h(x, y) = E[h(x, Y(V(t), x, y))],$$

where V is a Poisson process with parameter δ_∞^{-1}, defines a strongly continuous contraction semigroup $S(t)$ on $C(E)$. The generator B of $S(t)$ is the bounded linear operator

$$B = \delta_\infty^{-1}(Q - I),$$

where Q is defined on $C(E)$ by $Qh(x,y) = h(x, y + \delta_\infty c(x,y))$. Finally,

$$\lim_{k\to\infty} \sup_{(x,y)\in E} |Q^k h(x,y) - h(x,0)| = 0, \quad h \in C(E).$$

Theorem A.3. *For $N = 0, 1, \dots$, let $(Z^N(k), k = 0, 1, \dots)$ be a Markov chain in a metric space E_N with transition function $p_N(z, A)$ and denote the 1-step transition w.r.t to a function f by $\mathbb{E}_z[f(Z^N(1))] = \int f(z')p_N(z, dz')$. Let $\Phi_N : E_N \to K$ and $\Psi_N : E_N \to H$ be Borel measurable, define $X^N(k) = \Phi_N(Z^N(k))$ and $Y^N(k) = \Psi_N(Z^N(k))$ for each $k \geq 0$. Let $\varepsilon_N > 0$, $\delta_N > 0$ and assume $\lim\limits_{N\to\infty} \frac{\varepsilon_N}{\delta_N} = 0$, as well as $\lim\limits_{N\to\infty} \delta_N = \delta_\infty \in [0, \infty)$.*
Let each of the functions $a : K \times \mathbb{R}^n \to \mathbb{R}^m \otimes \mathbb{R}^m$, $b : K \times \mathbb{R}^n \to \mathbb{R}^m$ and $c : K \times \mathbb{R}^n \to \mathbb{R}^n$ be continuous and suppose that, for $i, j = 1, \dots, m$ and $l = 1, \dots, n$,

$$\varepsilon_N^{-1}\mathbb{E}_z[X_i^N(1) - x_i] = b_i(x,y) + o(1), \tag{A.8}$$

$$\varepsilon_N^{-1}\mathbb{E}_z[(X_i^N(1) - x_i)(X_j^N(1) - x_j)] = a_{ij}(x,y) + o(1), \tag{A.9}$$

$$\varepsilon_N^{-1}\mathbb{E}_z[(X_i^N(1) - x_i)^4] = o(1), \tag{A.10}$$

$$\delta_N^{-1}\mathbb{E}_z[Y_l^N(1) - y_l] = c_l(x,y) + o(1), \tag{A.11}$$

$$\delta_N^{-1}\mathbb{E}_z[(Y_l^N(1) - \mathbb{E}_z[Y_l^N(1)])^2] = o(1) \tag{A.12}$$

as $N \to \infty$, uniformly in $z \in E_N$, where $x = \Phi_N(z)$ and $y = \Psi_N(z)$.

Let

$$G = \frac{1}{2}\sum_{i,j=1}^m a_{ij}(x,0)\frac{\partial}{\partial x_i \partial x_j} + \sum_{i=1}^m b_i(x,0)\frac{\partial}{\partial x_i} \tag{A.13}$$

and assume that the closure of $\{(f, Gf) : f \in C^2(K)\}$ is single-valued and generates a Feller semigroup $U(t)$ on $C(K)$, corresponding to a diffusion process X in K. Suppose further that c satisfies the conditions of Lemma A.1 if $\delta_\infty = 0$ or of Lemma A.2 if $\delta_\infty > 0$. Then the following conclusions hold:
a) If $X^N(0) \Rightarrow X(0)$ in K, then $X^N([\bullet/\varepsilon_N]) \Rightarrow X$ in $D_K([0,\infty))$.
b) If $\{t_N\} \subset [0,\infty)$ satisfies $\lim\limits_{N\to\infty} t_N = \infty$, then $Y^N([t_N/\delta_N]) \Rightarrow 0$ in H.

Lemma A.4. *If $\lim\limits_{N\to\infty} \varepsilon_N = 0$ and the first condition (A.8) ist satisfied, then it is sufficient to check*

$$\varepsilon_N^{-1}\mathbb{E}_z[(X_i^N(1) - \mathbb{E}_z[X_i^N(1)])(X_j^N(1) - \mathbb{E}_z[X_j^N(1)])] = a_{ij}(x,y) + o(1), \text{ for all } i,j = 1,\dots,m, \tag{A.14}$$

for (A.9) to be satisfied. Additionally, it is sufficient to check

$$\varepsilon_N^{-1}\mathbb{E}_z[(X_i^N(1) - \mathbb{E}_z[X_i^N(1)])^4] = o(1), \text{ for all } i = 1, \dots, m, \tag{A.15}$$

to obtain (A.10).

C Diffusion limit

We define the state space for the diffusion process X

$$K = \{x = (x_{ij})_{(i,j)\in[J]} \in [0,1]^{r_A r_B - 1} : \sum_{(i,j)\in[J]} x_{ij} \le 1\}. \tag{A.16}$$

The generator for X is given by

$$\mathscr{L} = \sum_{(i,j)\in[J]} \frac{1}{2}\Bigg[\sum_{(k,l)\in[J]} x_{ij}(\delta_{ik}\delta_{jl} - x_{kl})\frac{\partial}{\partial x_{kl}} + b_{ij}(x)\Bigg]\frac{\partial}{\partial x_{ij}}, \tag{A.17}$$

where

$$b_{ij}(x) = \theta_A \sum_k x_{kj}(P_{ki}^A - \delta_{ik}) + \theta_B \sum_l x_{il}(P_{lj}^B - \delta_{lj}) - \rho(x_{ij} - x_{i\bullet}x_{\bullet j}).$$

With the help of Theorem A.3, we can observe the following facts.

Lemma A.5. *Let X be the diffusion process associated with the generator (A.17) and state space K, defined in (A.16).*
1) If $X^N(0) \Rightarrow X(0)$ in K, then $X^N([2N_e\bullet]) \Rightarrow X$ in $D_K([0,\infty))$.
2) If $\{t_N\} \subset [0,\infty)$ satisfies $\lim_{N\to\infty} t_N = \infty$, then $Y^N([t_N]) \Rightarrow 0$.

Remark A.6. *(Y approximately 0 in the slow time scale) For each $t > 0$, we can conclude by b) that*

$$Y^N([2N_e t]) \Rightarrow 0 \text{ as } N \to \infty,$$

since $\{t_N\} \subset [0,\infty)$ with $t_N := (t/\varepsilon_N)\delta_N = 2N_e t$ satisfies $\lim_{N\to\infty} t_N = \infty$.

Proof. 1.)
The application of the Theorem A.3 requires the verification of moment conditions on $X^N = \Phi_N(Z^N)$ and $Y^N = \Psi_N(Z^N)$. The moment conditions can be derived from the following observations.

$$\mathbb{E}_z[P'_{\alpha ij} - P_{\alpha ij}] = P_{\alpha ij}^{***} - P_{\alpha ij}, \tag{A.18}$$

which follows from the properties of the multinomial distribution.
The aim is now to get the parameters, related to the mechanisms, into the game. Then we can use the scaling behavior we assumed.

We obtain

$$P^{***}_{\alpha ij} - P_{\alpha ij} = P^{***}_{\alpha ij} - P^{**}_{\alpha ij} + P^{**}_{\alpha ij} - P^{*}_{\alpha ij} - r_N D^N_{\alpha ij}, \tag{A.19}$$

since

$$P^{*}_{\alpha ij} = \sum_{k,l,m,n} \theta^N_{ij;kl,mn} P_{\alpha kl} P_{\alpha mn}$$

$$= (1 - r_N) P_{\alpha ij} + r_N P_{\alpha \bullet j} P_{\alpha i \bullet} = P_{\alpha ij} - r_N D^N_{\alpha ij},$$

with

$$D^N_{\alpha ij} = P_{\alpha ij} - P_{\alpha i\bullet} P_{\alpha \bullet j}.$$

and $P_{\alpha i\bullet} = \sum_j P_{\alpha ij}$, $P_{\alpha \bullet j} = \sum_i P_{\alpha ij}$. We used the fact $\sum_{k,l} P_{\alpha kl} = 1$. As the next step, we resolve the expression $P^{***}_{\alpha ij}$.
Recalling (A.1), (A.2), multiplication directly implies

$$P^{***}_{\alpha ij} = P^{**}_{\alpha ij} - \sum_m u^N_{im} P^{**}_{\alpha ij} + \sum_k u^N_{ki} P^{**}_{\alpha kj} - \sum_n v^N_{jn} P^{**}_{\alpha ij} + \sum_l v^N_{lj} P^{**}_{\alpha il} + O(N_e^{-2}) \tag{A.20}$$

Note that $\mathbb{E}_z[P^{'}_{ij} - P_{ij}] = \sum_\alpha \xi_\alpha \mathbb{E}_z[P^{'}_{\alpha ij} - P_{\alpha ij}]$ and recall $\sum_\alpha \xi_\alpha m_{\alpha\beta} = \xi_\beta$. From this, the required moment conditions (A.8) and (A.9) can be derived by setting $d_{\alpha ij} = 0$, using the assumed parameter assumptions, multiplying with $2N_e$ and an application of Lemma A.4.

The third condition (A.10) follows from Lemma A.4, in particular (A.15), and the moment properties of the binomial distribution.
We define

$$c_{\alpha ij}(P, d) := \sum_\beta m_{\alpha\beta} d_{\beta ij} - d_{\alpha ij}. \tag{A.21}$$

The fact that c satisfies the conditions of Lemma A.2 can be deduced with the same argumentation as in [2] (p. 111).
Consider

$$\mathbb{E}_z[d^{'}_{\alpha ij} - d_{\alpha ij}] = P^{***}_{\alpha ij} - \sum_\beta \xi_\beta P^{***}_{\beta ij} - d_{\alpha ij}$$

$$= \sum_\gamma m_{\alpha\gamma} P_{\gamma ij} - \sum_\gamma \sum_\beta \xi_\beta m_{\beta\gamma} P_{\gamma ij} - d_{\alpha ij} + O(N_e^{-1})$$

$$= \sum_\beta m_{\alpha\beta} d_{\beta ij} - d_{\alpha ij} + O(N_e^{-1}).$$

An application of Lemma A.4 and $\sum_\beta \xi_\beta m_{\beta\gamma} = \xi_\gamma$ yield condition (A.11).

$$\mathbb{E}_z[(d^{'}_{\alpha ij} - \mathbb{E}_z[d^{'}_{\alpha ij}])^2] = \mathrm{Var}_z(d^{'}_{\alpha ij}) = \mathrm{Var}_z(P^{'}_{\alpha ij} - P^{'}_{ij})$$

$$\leq 2\mathrm{Var}_z(P^{'}_{\alpha ij}) + 2\mathrm{Var}_z(P^{'}_{ij}) = 2(2N_\alpha)^{-1}P^{***}_{\alpha ij}(1-P^{***}_{\alpha ij}) + 2(2N_\alpha)^{-1}\sum_\gamma \xi_\gamma P^{***}_{\gamma ij}(1-P^{***}_{\gamma ij}).$$

implies (A.12).
Ethier argued in [4] and [5] that the closure of the generator (A.17) generates a Feller Semigroup. This concludes that we can apply Theorem A.3.

2.)
See 1.) and conclusion b) of Theorem A.3. □

Note that we can use the more convenient form

$$K_c = \{x = (x_{ij})_{i\in[r_A],j\in[r_B]} \in [0,1]^{r_A r_B} : \sum_{i\in[r_A],j\in[r_B]} x_{ij} = 1\}, \tag{A.22}$$

for the state space and express the generator as

$$\mathscr{L} = \sum_{i\in[r_A],j\in[r_B]} \frac{1}{2}\left[\sum_{k\in[r_A],l\in[r_B]} x_{ij}(\delta_{ik}\delta_{jl} - x_{kl})\frac{\partial}{\partial x_{kl}} + b_{ij}(x)\right]\frac{\partial}{\partial x_{ij}}. \tag{A.23}$$

The latter representation is often used in the context of population genetics.

D Proof of Lemma 1

Fix a $N \in \mathbb{N}$, denote the relative frequencies for Z^N by $P_{\alpha ij}$ and fix α, i, j. $\sum_{ij} P^{**}_{\alpha ij} = 1$ together with the parameter assumption imply $0 < P^{***}_{\alpha ij} < 1$ for all $(P_{\alpha ij})_{\alpha,i,j} \in E_N$. By the properties of the multinomial distribution of $(P^{'}_{\alpha ij})_{\alpha ij}$ in (A.7) we can observe that Z^N is aperiodic and irreducible. This concludes the statement.

E Proof of Lemma 3

The proof of Lemma 3 uses the objects of the derivations and statements of Theorem A.3.

For every $N \geq 1$, $\mu_N \circ \Phi_N^{-1} \in \mathcal{P}(K)$. Since K is compact, we choose a subsequence $(\mu_{N'} \circ \Phi_{N'}^{-1})_{N'}$ which converges to a weak limit point $\mu_0 \in P(K)$. Rename the subsequence to $(\mu_N \circ \Phi_N^{-1})_N$. Introduce $\pi^{'}_N : C(K) \to \overline{C}(E_N)$ by $(\pi^{'}_N f)(z) = f(\Phi_N(z))$, since K is compact.
Choose $T > 0$, $f \in C(K)$ and $0 \leq t \leq T$, then we can write

$$\left|\int U(t)f\,d\mu_0 - \int f\,d\mu_0\right|$$

$$\leq \left|\int U(t)f\,d\mu_0 - \int \pi^{'}_N U(t)f\,d\mu_N\right| + \left|\int (\pi^{'}_N U(t)f - T_N^{[2N_e t]}\pi^{'}_N f)\,d\mu_N\right|$$

$$+\left| \int T_N^{[2N_e t]} \pi_N' f \, d\mu_N - \int f \, d\mu_0 \right|.$$

From this we can conclude

$$\int U(t) f \, d\mu_0 = \int f \, d\mu_0$$

for $f \in C(K)$. Since $C(K)$ is separating and the unique stationary distribution μ is unique, we know that every weak limit point for $\mu_N \circ \Phi_N^{-1}$ coincides with μ and therefore the sequence converges weakly to μ. This shows the convergence of the discrete stationary distributions to the stationary distribution of the diffusion process. We also know $Y^N \Rightarrow 0$ as $N \to \infty$, that means that the derivations from the global frequencies within the subpopulations vanish. This immediately implies the statement of the Lemma by the form of $\mathcal{P}$.

References

[1] Ethier SN, Nagylaki T. Diffusion approximations of the two-locus Wright-Fisher model. J Math Biol. 1989; 27: 17–28.

[2] Nagylaki T. The strong-migration limit in geographically structured populations. J Math Biol. 1980; 9: 101–114.

[3] Ethier SN, Kurtz TG. Markov Processes: Characterization and Convergence. Wiles Series in Probability and Statistics, John Wiley & Sons, Inc., New York 2005.

[4] Ethier SN. A class of degenerate diffusion processes occuring in population genetics. Comm Pure Appl Math. 1976; 29: 483–493.

[5] Ethier SN. A limit theorem for two-locus diffusion models in population genetics. J Appl Probab. 1979; 16: 402–408.
